# Editorial Comment: Does pre-operative urodynamics lead to better outcomes in management of urinary incontinence in women? A linked systematic review and meta-analysis

**DOI:** 10.1590/S1677-5538.IBJU.2020.03.09

**Published:** 2020-02-20

**Authors:** KY Lor, M Soupashi, M Abdel-Fattah, A Mostafa, Cássio L. Z. Riccetto

**Affiliations:** 1 University of Aberdeen Aberdeen UK University of Aberdeen, Aberdeen, UK; 2 University of Aberdeen Aberdeen UK University of Aberdeen, Aberdeen, UK; 1 Faculdade de Ciências Médicas da Universidade Estadual de Campinas – UNICAMP Divisão de Urologia Feminina CampinasSP Brasil Divisão de Urologia Feminina - Faculdade de Ciências Médicas da Universidade Estadual de Campinas – UNICAMP, Campinas, SP, Brasil

## COMMENT

The usefulness of urodynamics in the evaluation of women with urinary incontinence is a recurring theme in the literature. Current is considered optional in index patients with typical stress incontinence (1) particularly if the first option is for physical therapy treatment. In the initial approach of patients with overactive bladder, whether wet or dry, is still considered to be expendable. On the other hand, its indication in patients with relapsed stress incontinence and mixed urinary incontinence is consensual (2). It is also found that few studies have included cost-effectiveness analysis among their outcomes. In this context, the authors presented three correlated systematic reviews and meta-analyzes of randomized controlled trials (RCTs) to compare the exclusive clinical versus the urodynamic use in three clinical scenarios: clinical pre-treatment of urinary incontinence, before surgical treatment of stress urinary incontinence and before invasive treatment of overactive bladder. Women with severe pelvic organ prolapse, previous continence surgery and neuropathic bladder were excluded from the analysis. Patient-reported and objective success post-treatment were the primary outcomes assessed and the secondary outcomes were adverse events, quality of life, sexual function and health economic measures (3).

Four RCTs compared urodynamics versus clinical evaluation only prior to non-surgical management of UI. Treatment consisted of. Meta-analysis of 150 women showed no evidence of significant difference in the patient-reported (P = 0.520, RR: 0.91, 95% Cl 0.69–1.21, I2 = 0%) and objective success rates (P = 0.470, RR: 0.87, 95% Cl 0.59-1.28, I2 = n / a) between pelvic floor muscle training alone compared to pelvic floor muscle training with pharmacological therapy. Seven RCTs evaluated surgical management of SUI. The majority of women underwent mid-urethral tape procedures (retropubic or transobturator approach). Meta-analysis of 1149 women showed significant difference in patient-reported (P = 0.850, RR: 1.01, 95% CI 0.88-1.16, I2 = 53%) and objective success between groups (P = 0.630, RR: 1.02, 95% CI 0.95 - 1.08, I2 = 28%). There was no significant difference in incidence of voiding dysfunction, again urgency, and urinary tract infection between groups. No RCTs have been identified for invasive management of OAB. The authors concluded that there is limited evidence that routine urodynamics prior to non-surgical management of urinary incontinence or surgical management of stress urinary incontinence did not correlate to improvement in treatment outcomes, when compared to exclusive clinical evaluation. They also see the need for new well-designed clinical trials to evaluate the clinical and cost-effectiveness of routine urodynamics prior to surgical management of stress urinary incontinence and overactive bladder.

The multifactorial pathophysiological aspect of female urinary incontinence represents a challenge for any attempt to standardize its assessment and even treatment. Urodynamics is still the most reliable instrument for specialized study of lower urinary tract symptoms. An individualized approach is the current trend to determine its utility in specific patients.

## References

[1] 1. Lightner DJ, Gomelsky A, Souter L, Vasavada SP. Diagnosis and Treatment of Overactive Bladder (Non-Neurogenic) in Adults: AUA/SUFU Guideline Amendment 2019. J Urol. 2019;202:558-63. 10.1097/JU.0000000000000309 31039103

[2] 2. Kobashi KC, Albo ME, Dmochowski RR, Ginsberg DA, Goldman HB, Gomelsky A, et al. Surgical Treatment of Female Stress Urinary Incontinence: AUA/SUFU Guideline. J Urol. 2017;198:875-83. 10.1016/j.juro.2017.06.061 28625508

[3] 3. Serati M, Tarcan T, Finazzi-Agrò E, Soligo M, Braga A, Athanasiou S, Balzarro M. The bladder is an unreliable witness: The case for urodynamic investigations in female stress urinary incontinence. Eur J Obstet Gynecol Reprod Biol. 2020;244:35-7. 10.1016/j.ejogrb.2019.10.046 31731022

